# Parents' Views on Family Care at a Neonatal Intensive Care Unit — A Qualitative Study

**DOI:** 10.1111/scs.70306

**Published:** 2026-08-19

**Authors:** Linea Grothe Larsen, Suzanne Forsyth Herling, Sanne Allermann Beck, Anne Brødsgaard, Jos M. Latour, Rikke Louise Stenkjaer

**Affiliations:** ^1^ Department of Pediatrics; Neonatal Intensive Care Unit Central and West Zealand Hospital Slagelse Denmark; ^2^ The Neuroscience Centre Rigshospitalet, University of Copenhagen Copenhagen Denmark; ^3^ University of Copenhagen Copenhagen Denmark; ^4^ Department of Neonatal and Pediatric Intensive Care Rigshospitalet, Copenhagen University Hospital Copenhagen Denmark; ^5^ Department for Health and Society, Institute of People and Technology Roskilde University Roskilde Denmark; ^6^ Department of Nursing and Health, Institute of Public Health Aarhus University Aarhus Denmark; ^7^ Department of Gynecology and Obstetrics Copenhagen University Hospital, Amager Hvidovre, Capital Region of Denmark Denmark; ^8^ Department of Pediatrics and Adolescent Medicine Copenhagen University Hospital, Amager Hvidovre, Capital Region of Denmark Denmark; ^9^ School of Nursing and Midwifery, Faculty of Health University of Plymouth Plymouth UK

**Keywords:** family care, family‐centred care, family‐integrated care, neonatology, parental experiences, quality of healthcare

## Abstract

**Purpose:**

The current study aims to explore parents' views on family care at a neonatal intensive care unit.

**Methods:**

We conducted a qualitative exploratory study with a descriptive approach based on open‐ended written narrative responses from the Danish version of EMpowerment of PArents in The Intensive Care—Neonatology questionnaire. The study was conducted at a Danish level IV NICU working with family‐centred care and family integrated care. The data was analysed using inductive content analysis by Graneheim and Lundman. The Standards for Reporting Qualitative Research checklist was followed.

**Main Findings:**

In total, 311/727 parents responded to the distributed questionnaire. Of those, 210 responded to the open‐ended questions. While general expressions of satisfaction and praise were noted, the qualitative analysis focused on responses that included more elaborated comments. We generated two themes, *Challenges in communication and recognition in NICU care* and *Balancing family needs and support systems in NICU.* Communication with nurses and physicians included both positive experiences and areas for improvement, with some parents experiencing confusion and insecurity. Emotional support, privacy and the opportunity for bonding were often overlooked, leaving parents feeling excluded from the care process. Parents reported a lack of individualized care that considered their unique needs as a family.

**Conclusion:**

The findings underscore the need for improved communication, better parental involvement in care decisions, and more attention to emotional and relational support. Addressing these areas could enhance the overall neonatal experience and the needs of infants and their families, ultimately improving care quality and satisfaction.

## 1 Background

Advancements in neonatology in the last decades have led to increased survival rates and better outcomes for infants in the neonatal intensive care unit (NICU) [1, 2]. Besides the technical advancements, the parental role has evolved from being mainly observers to active participants in caring for the infant [1, 3]. Earlier beliefs held that parental visits could disrupt effective care and consequently hinder the recovery of the infant [3, 4]. However, studies have demonstrated that a lack of parental visits caused severe emotional distress and long‐term psychological effects among infants extending into adolescence and adulthood [4, 5]. Later studies found that skin‐to‐skin contact strengthened bonding and reduced pain and hormonal stress in preterm infants [6], prompting a more holistic, family‐inclusive approach with unrestricted visiting hours [1, 4]. However, NICU admission can be emotionally challenging for parents, who may experience increased levels of stress, anxiety and emotional distress [7, 8]. These responses are often related to concerns about the infant's health and survival, the sensory environment of the NICU such as noise and alarms from medical equipment and disruptions to expected parental roles [9]. Providing emotional support through consistent and clear communication, psychological support and facilitating parental presence and involvement during hospitalization is therefore essential [10].

Family‐centred care (FCC) has emerged as an approach to address challenges and needs essential to high‐quality care [11]. FCC aims to achieve a partnership between patients, families and healthcare professionals (HCPs) based on four principles: (1) respect and dignity, (2) information sharing, (3) participation and (4) collaboration [12]. Identifying the most effective FCC interventions and implementing them has proven a difficult challenge as it is hampered by a lack of consensus on the most appropriate outcomes [13, 14]. Despite the challenges, research on FCC interventions has grown over the past 15 years. Comphrehensive models of NICU care that involve partnerships with current and former NICU families in the design and delivery of care are supported by a growing evidence base [15]. One such model, Family Integrated Care (FICare) is an extension of FCC, in which parents are empowered and supported to take the role of primary caregivers in the NICU. FICare includes five key principles: (1) partnership with families, (2) empowerment, (3) well‐being, (4) culture and (5) environment. FICare has demonstrated improved short‐term outcomes such as improved infant weight gain, high frequency exclusive breast milk feeding at discharge and reduces length of stay [16, 17]. Organizing care following the principles of FICare benefits parents as it decreases parental stress, supports their parental role, strengthens family function during admission and reduces length of stay [16, 18]. This study aimed to explore parents' qualitative views on family care in a NICU. Understanding their perspectives can improve the quality of care, help tailor interventions to meet the needs of infants and parents and guide HCPs and healthcare services.

## 2 Materials and Methods

### 2.1 Study Design

We conducted a qualitative exploratory study with a descriptive approach based on open‐ended written narrative responses from the Danish version of EMpowerment of PArents in The Intensive Care—Neonatology (EMPATHIC‐N) questionnaire [19]. The researchers involved in this study were nurses with experience in intensive care or neonatal practices. None were involved in clinical care in the study NICU during the study period. All had experience with qualitative research methods, although with varying degrees of proximity to the field [20, 21].

### 2.2 Setting

The study was performed at a 33‐bedded level IV NICU with around 1200 admissions annually. The NICU has its own intake area, and these children remain in the unit until discharge. The case mix of the children therefore varies and includes premature infants from 23 weeks gestational age, new‐born infants with congenital diseases, organic defects, birth‐related complications, infections or other issues in the neonatal period. FCC and FICare have been fundamental principles in the unit for the past 20 years and all newly hired nurses receive 4 h of classroom training in the FCC and FICare principles and are supervised bedside by a mentor. In 2015, the unit became Newborn Individualized Developmental Care (NIDCAP) certified. The patient‐to‐nurse ratio is 2:1, depending on the complexity of the infant. Infants are cared for in double‐occupancy rooms with a bed for one parent close to the infant. The unit has unrestricted access for parents and other relatives.

### 2.3 Ethical Considerations

Approval to collect and store data was in accordance with the Danish Agency for Data Protection and the Helsinki Declaration [22]. Further ethical approval was not required due to Danish law. Parents gave consent to participate when completing the questionnaire and were assured of confidentiality, anonymization of data and publication of the results. For this article, data were anonymized.

### 2.4 Data

The validated EMPATHIC‐N questionnaire measures the delivered care perceived by parents with an infant admitted to a NICU [19]. EMPATHIC‐N was developed based on the perspectives of parents and NICU staff and embedded in the theory of FCC [19]. The 57‐item questionnaire explores parents' experiences within five domains: (1) information, (2) care and treatment, (3) organization, (4) parental participation and (5) professional attitude, plus five open‐ended questions. The quantitative results of the survey were previously published by Weis et al., which showed that parent satisfaction was high and included the translation and validation of the EMPATHIC‐N in Danish [23]. Our study analysed the qualitative results of the survey in form of narrative responses to the open‐ended questions, where parents described their experiences with the NICU unit and staff, admission, hospitalization, transfers/discharge and overall experiences (Table 1).

**TABLE 1:** The five open‐ended questions from EMPATHIC‐N‐DK.

No.	Question
1	‘If you would like to elaborate, you can write it below’. Based on: – Two statements rated on a 6‐point Likert scale (1 = Strongly Disagree, 6 = Strongly Agree): We would recommend this neonatal unit to others in a similar situation. If we were in the same situation again, we would want to return to this neonatal unit. – One statement rating nurses and doctors on a 10‐point Likert scale (1 = Poor, 10 = Excellent): What score would you give for the overall hospitalization experience?
2	‘Your experiences regarding ADMISSION to the neonatal unit’
3	‘Your experiences DURING the hospitalization’
4	‘Your experiences regarding TRANSFER/DISCHARGE’
5	‘Your OVERALL experiences’

Data originated from 727 invited parents of preterm or ill newborn infants who had been cared for at the NICU for at least 48 h and could read Danish. Of those, 311 responded. Parents of multiple births received only one questionnaire. Exclusion criteria were parents of infants who died in the unit as they were not eligible to participate. During admission, parents signed a statement and provided their email if they agreed to be contacted after discharge to share feedback on their experiences. Two weeks after discharge, emails were sent to one of the parents containing information about the study and a link to the online questionnaire. In case of no response, one email was sent to one of the parents 2 weeks after the first email repeating the invitation. The information clarified that answering the questionnaire was considered consent to participate in the study. The EMPATHIC‐N‐DK was set up in a secure database using an Enalyzer survey application. Data were collected between June 2017 and November 2019.

### 2.5 Data Analysis

A qualitative content analysis was conducted based on Graneheim and Lundman [24]. This framework offers a structured approach to systematically analyse qualitative content including both manifest and latent meanings differing mainly in depth and level of abstraction [25].

All responses were read several times to grasp the whole, and each respondent's answers to the five open‐ended questions formed the units of analysis. The first author condensed meaning units, abstracted them and assigned codes, which were then organized into subcategories and categories, supported by quotes. These were discussed and refined by the first, second and last author. Themes were then developed by the whole author group interpreting the latent content of categories through reflection and dialogue. Trustworthiness was enhanced through researcher triangulation for credibility, detailed descriptions of the setting for transferability, direct quotes to support confirmability and repeated discussions to ensure dependability [26]. The Standards for Reporting Qualitative Research guidelines were followed to ensure reporting transparency [27], and NVivo 14 was used for data organization.

## 3 Findings

The questionnaire was completed by 311 parents (Table 2). In total 210/311 (68%) responded to the open‐ended questions. Of those, 123 were completed by mothers, 21 by fathers, 65 by both parents and 1 by two mothers. In total, there were 674 qualitative responses. In general, the parents were highly satisfied with the care and treatment that saved the life of their infants, and many stated their gratitude towards the treatment and care. However, we only analysed responses that had more content than general satisfaction and praise (e.g., ‘Good’, ‘OK’, ‘See previous comment’) to gain insight into how family care could be improved in the NICU. From these 589 responses, we generated two themes: (1) Challenges in communication and recognition in NICU care and (2) Balancing family needs and support systems in NICU. The themes were based on five categories and eight subcategories (Table 3), which were supported by quotes (Table 4).

**TABLE 2:** Characteristics of parents and infants.

Parents (*n* = 311)		
Cultural background (*n* (%))		
Danish	294 (94.5%)	
Other	17 (5.5%)	
Education level (*n*/%)	Mothers *n* = 309	Fathers *n* = 302
Elementary school or less	7 (2.2%)	14 (4.5%)
High school degree	23 (7.4%)	25 (8.0%)
Occupational education	11 (3.5%)	39 (12.5%)
Short secondary (2–3 years)	30 (9.6%)	31 (10.0%)
Medium length (3–4 years)	96 (30.9%)	61 (19.6%)
Long secondary (4–6 years or longer)	139 (44.7%)	121 (38.9%)
Not applicable	2 (0.6%)	11 (3.5%)
Infants (*n* = 324)		
Gender boy (*n* (%))	202 (62.3%)	
Gestational age $w^{+d}$(mean/min‐max)	$35^{+5}/23^{+1}$‐ $42^{+2}$	
Birth weight g (mean/min‐max)	2611/470–4900	
Length of stay d (mean/min‐max)	13/2–113	
Days on ventilator *n* = 98 (mean/min‐max)	6.5/1–85	
Days on CPAP *n* = 189 (mean/min‐max)	9/1–102	
Diagnosis (*n* (%))		
Preterm	125 (38.6%)	
Common neonatal complications	144 (44.4%)	
Congenital cardiac defect	28 (8.6%)	
Congenital abdominal defect	27 (8.3%)	

*Note:* In some cases, parent education was not answered; common neonatal complications include for eksampel asphyxia, cerebral complications, sepsis or jaundice.

Abbreviations: d, days; g, grams; N, number; w, weeks.

**TABLE 3:** Themes, categories and subcategories.

Theme	Category	Subcategory
1: Challenges in communication and recognition in NICU care.	1.1: A lurking insecurity.	1.1.1: Trust in healthcare professionals.
		1.1.2: Lack of individualized, situation‐specific communication
	1.2: Struggle to be recognized as partners in care.	1.2.1: Overwhelming sense of responsibility.
		1.2.2: Feeling overlooked in a high‐stakes environment.
2: Balancing family needs and support systems in NICU.	2.1: Desire for alignment in priorities.	2.1.1: Consolidating the new family.
		2.1.2: Impersonalized care.
	2.2: Parental dependency and support from their partner.	2.2.1: Mutual reliance in times of crisis.
	2.3: Lack of attention to the mental state of parents.	2.3.1: Limited facilities for privacy and family comfort.

**TABLE 4:** Example of the analysis process.

Theme	Challenges in communication and recognition in NICU care
Category	Struggle to be recognized as partners in care
Subcategory	Feeling overlooked in a high‐stakes environment
Code	To feel seen and prioritized
Condensed meaning unit	Parents of children that aren't very sick feels under‐prioritized and somewhat forgotten in the staff's daily routines
Meaning unit	‘Our child wasn't among the very sick, so resources were often prioritized elsewhere (which is completely understandable). However, we sometimes felt under‐prioritized and somewhat forgotten in the staff's very busy daily routines.’ (77, Both parents)

### 3.1 Theme 1: Challenges in Communication and Recognition in NICU Care

The general narrative in this theme encompasses the difficulties parents face in terms of communication with nurses and physicians and the struggle to be acknowledged as integral partners in the care process.

#### 3.1.1 Category 1.1: A Lurking Insecurity

During the stay, parents felt a subtle but persistent unease. Grateful for the life‐saving care, they noticed inconsistencies in treatment and observations that gradually eroded their confidence and highlighted their need for personalized care. Over time, this uncertainty extended beyond the clinical situation, leaving parents unsure about the quality of care and their role within the setting.

##### 3.1.1.1 Subcategory 1.1.1: Trust in Healthcare Professionals

Most parents described the hospitalization as a difficult and chaotic time, where the presence of professional and skilled nurses and physicians created a sense of security and safety:

We generally felt very well treated and are convinced that the doctors and nurses were extremely competent. (215, Both parents)

However, perceived unprofessionalism instantly sparked insecurity among parents:

(…) the less experienced nurses made us feel a bit uneasy because they made mistakes in the care/treatment and talked about internal matters concerning the department in front of us in the hospital room. (24, Mother)

Optimally, parents wanted nurses and physicians who were professional, considerate and capable of managing their infants' conditions throughout the hospitalization. As infants shifted from needing highly intensive care to more regular care in the unit, not all parents felt relieved by the progress as the care conditions changed and were perceived to be less competent. In fact, some parents felt uneasy that less experienced HCPs might handle procedures or care, while more critically ill infants were cared for by the more experienced HCPs:

(…) this was also where we were ‘exposed’ to ventilator staff, nurses, doctors, and lab technicians who were still in training. I really felt like we were being used for practice because we had a healthy child. It was extremely uncomfortable. (59, Mother)

Few parents felt they had to shield their infant and highlighted a distrust towards HCPs and a fear and insecurity that the quality of care was not the same standard throughout the whole hospitalization period. Parents needed to feel assured about the care their infant was receiving as this provided the best possible foundation for their later transition to life at home together as a family.

##### 3.1.1.2 Subcategory 1.1.2: Lack of Individualized, Situation‐Specific Communication

Parents felt supported and appreciated when information was shared in a clear, timely and sensitive manner. However, some parents experienced that information was provided in a way that did not always consider individual needs or specific circumstances. When information was not adapted to the level of understanding or mental capacity at the time, it led to a sense of information overload. The volume and timing of information influenced the ability to process and act on it:

When you've just given birth and then get admitted, you don't really have the energy or headspace to properly take in what's being said. I honestly don't know what was said that night [the night the baby was born]. (93, Both parents)

At the same time, some parents felt that some information was under‐shared, making it difficult to understand and meet both spoken and unspoken expectations of nurses or physicians. When internal expectations and ‘house rules’ were not explicitly communicated, parents had to rely on their realizations and interpretations of what was required or expected of them:

We were directly scolded by a nurse at the neonatal unit because no parent had stayed with our baby that first night—but we hadn't been told that it was even an option, let alone an expectation. (154, Mother)

Information sharing took place primarily on the nurses' or physicians' terms and was often limited to the immediate situation in the department and internal rules and regulations in the organization. Some parents experienced that they were not informed about routine changes, such as new levels of (less) observation when their infant was weaned off mechanical ventilation:

We had to figure out on our own that when a baby comes off the ventilator, the staff no longer necessarily needs to be in the room. We eventually realized that, but it really would have been helpful if someone had explained that this is how the house rules work… (224, Both parents)

The overall communication style reflected a one‐way flow of information, where some nurses and physicians assumed parents understood internal ward rules and where parents were expected to absorb and act on the information provided. The organizational structure appeared to prioritize standardized information delivery over dialogue‐based and situation‐specific communication.

#### 3.1.2 Category 1.2: Struggle To Be Recognized as Partners in Care

While most parents felt empowered and appreciated the guidance nurses and physicians provided, others felt disregarded or micromanaged as if their autonomy as primary caregivers were suspended. The extent to which parents were involved shaped their overall perception of partnership in care.

##### 3.1.2.1 Subcategory 1.2.1: Overwhelming Sense of Responsibility

The balance between professional authority and parental involvement varied across different care experiences. When guidance was offered in a way that respected their autonomy, parents felt empowered and included in decision‐making. However, some parents perceived a more controlling and paternalistic way of care, where nurses exerted authority over daily caregiving tasks:

The nurses were very kind and caring, but they did tend to be quite controlling. It often felt like we had to ask for permission for everything—even though it was our child. (55, Mother)

Structured routines supported the infant's well‐being, but rigid caregiving frameworks sometimes left parents feeling excluded and alienated as primary caregivers within the hospital's hierarchy. In contrast, most experienced a balanced approach, with nurses fostering their independence:

Thank you to all the nurses. You helped us incredibly much during the first days at the NICU and provided us with confidence to take care of our tiny new baby boy. (172, Mother)

Another aspect of the power dynamic appeared when delays or inconsistencies in care shifted added responsibility onto parents. Disrupted medication schedules often forced them into informal care manager roles, monitoring treatment and advocating for interventions. Repeatedly reminding staff about critical tasks, like medication timing, heightened their sense of powerlessness—they could not administer the medication themselves, but knew timely medications were vital for their infant's recovery:

There were also several instances of significant delays in our child's medication. At one point, we had to speak up—it had been 13 hours overdue! (201, Both parents)

These experiences reinforced the delicate balance between parental involvement and professional responsibility. While parents desired active participation, the necessity of constant vigilance to ensure proper care contributed to an overwhelming sense of responsibility.

##### 3.1.2.2 Subcategory 1.2.2: Feeling Overlooked in a High‐Stakes Environment

Some parents encountered situations where their emotional state and concerns were not fully acknowledged. When nurses and physicians failed to recognize parents' perspectives or expertise regarding their infant, the sense of partnership in care was compromised:

We didn't feel much understanding from the staff—our experience and concerns were dismissed, which unfortunately led to a sense of mistrust on our part. (319, Mother)

The prioritization of more critically ill infants within the unit was accepted by most parents, who understood the necessity of triaging care. However, when their own infant's needs were consistently deprioritized, it fostered a sense of invisibility:

Our child wasn't among the very sick, so resources were often prioritized elsewhere (which is completely understandable). However, we sometimes felt under‐prioritized and somewhat forgotten in the staff's very busy daily routines. (77, Both parents)

Sensing the overburdened nature of the staff contributed to an increased feeling of insecurity. The literal and figurative distance between parents and the nurses or physicians reinforced a sense of being left in the background rather than integral to the care process.

### 3.2 Theme 2: Balancing Family Needs and Support Systems in NICU

This theme highlights the importance of aligning care with parents' priorities, recognizing parents' dependence on their partners and supporting parents' mental well‐being, emphasizing the need for personalized and supportive care environments.

#### 3.2.1 Category 2.1: Desire for Alignment in Priorities

Nurses and physicians prioritized immediate care and stabilization, while parents, though concerned with clinical stability, also valued emotional bonding and family unity. Aligning these priorities was key to meeting both medical and emotional needs.

##### 3.2.1.1 Subcategory 2.1.1: Consolidating the New Family

Many parents acknowledged the nurses and physicians as supportive and strengthening of family unity during critical care experiences:

Our admission was very sudden, but despite the circumstances, we felt safe and were involved as much as possible. We felt that everyone did everything they could to save our daughter. (244, Mother)

From some parents' perspective, a successful hospitalization was not solely defined by medical treatment but also by the ability to prepare the infant and parents for everyday life after discharge. The parents saw breastfeeding as integral to their infant's development, recovery and long‐term well‐being. The lack of emphasis on breastfeeding support left some parents feeling that their role in their infant's immediate and long‐term care was underprioritized:

Even if it's not their main responsibility, the staff should generally be much better trained in breastfeeding support—especially when it comes to babies who've had a difficult start. It's unfortunate when a successful treatment for critical illness can't be followed up with successful breastfeeding. (183, Mother)

A few parents felt that breastfeeding was important for both their infant's development and their family's well‐being. When these broader aspects, shared goals and understandings between nurses/physicians and parents were not sufficiently prioritized, it created a discord between parental expectations and the hospital's focus.

##### 3.2.1.2 Subcategory 2.1.2: Impersonalized Care

Parents experienced a difference in perspective regarding their role in their infant's care. They appreciated when nurses or physicians acknowledged them as active partners and tailored parent involvement and care to the infant's specific needs. When this happened, parents felt included and part of the treatment process:

(…) the nurses and doctors were good at listening to us as parents—they involved us so that we could make a treatment plan together (…). (12, Both parents)

However, when care was structured around standardized routines rather than individual needs, parents felt like passive observers rather than primary caregivers. In some cases, they perceived that the department prioritized efficiency and clinical workflows over the family's emotional and practical needs, expressing disappointment with physicians who oriented themselves in the office and not at the bedside of the infant. Sometimes parents felt that their presence and concerns were secondary, and their infant was a mere case:

One could get the sense that we were little more than a case to them, and that was actually quite uncomfortable. (242, Father)

Additionally, the organization of care did not always support continuity for the family. Care often felt fragmented rather than part of a cohesive plan and it left some parents feeling unwanted, pushed around by the system and powerless in their situation:

After the surgery and time in intensive care, we were moved to the neonatal unit, where they talked about sending us back to the cardiac unit. But the heart specialist had told me that our son was finished with his heart treatment, and they didn't want us back there. Meanwhile, the neonatal doctor said we *should* go back to the cardiac unit. It was VERY confusing. (166, Mother)

The separation of mother and infant due to hospital logistics further complicated the parents' experience, making it difficult for them to maintain a sense of family unity and stability. Instead of prioritizing the family's ability to stay together, organizational routines dictated care practices that sometimes required parents to split up and be in multiple places simultaneously.

The pace and structure of hospital transitions reflected a prioritization of bed management and efficiency over the needs of individual families. When decisions were made quickly, parents felt that their need for stability and predictability was overlooked in favour of logistical considerations:

Things moved very quickly when the decision was made unexpectedly fast. As a result, there was very little time to prepare. (297, Mother)

While the healthcare system prioritized optimizing resources and ensuring timely transitions, admissions and discharges, parents valued a sense of continuity and respect for the need to feel prepared. The discrepancy between these perspectives contributed to feelings of uncertainty and lack of control. When transitions were managed with limited attention to the parental experience, it reinforced the notion that hospital workflows took precedence over family care.

#### 3.2.2 Category 2.2: Parental Dependency and Support From Their Partner

Parents relied on each other for support. In times of uncertainty, their bond became crucial for managing the emotional strain, offering reassurance and comfort. This mutual support helped them navigate fear and anxiety, providing stability in an unpredictable environment. The strength they drew from each other played a key role in their resilience through the experience.

##### 3.2.2.1 Subcategory 2.2.1: Mutual Reliance in Times of Crisis

The emotional turmoil caused by the uncertainty of their infant's survival intensified the parents' need for mutual support. Facing the possibility of loss created an overwhelming emotional burden, making their partner's presence and reassurance an essential anchor during the time of crisis. Parents expressed that their partner's support was not only a practical necessity but also a fundamental emotional lifeline, helping them navigate the distress and fear associated with their infant's illness and uncertainty about survival:

You really need support from your partner—especially when it's about a sick child and you're not even sure if you'll be bringing your baby home. (22, Mother)

In this context, the relationship between partners became a crucial source of strength, allowing them to share their anxieties, provide comfort and support each other through the unpredictable and emotionally exhausting experience of hospitalization.

#### 3.2.3 Category 2.3: Lack of Attention to the Mental State of Parents

The NICU environment placed a significant strain on parents' mental well‐being. While hospitalization was essential for their infant's care, the physical surroundings often became a tolerated necessity and not a space where they could fully function as a new family.

##### 3.2.3.1 Subcategory 2.3.1: Limited Facilities for Privacy and Family Comfort

Parents expressed that the absence of private spaces conflicted with their fundamental needs to bond as a new family. The constant presence of others, whether medical staff or other families, made it difficult to find moments for privacy and closeness:

The lack of private space constantly conflicted with our deep desire to be together as a new family (…). (62, Father)

The shared rooms intensified emotional vulnerability, as parents were continuously exposed to the joys and sorrows of other families, making it difficult to process and focus solely on their own experiences. The lack of natural daylight, restricted movement, and the overwhelming medical atmosphere contributed to a sense of entrapment:

And the single screen that separated us from the other family also blocked out daylight and any view of the ‘real world’ (…). It honestly felt like being locked up in a prison—at times, it felt like torture. (195, Mother)

Despite these challenges, some parents found solace in support services such as yoga or psychological counselling, which offered brief moments of relief from the mental burden of hospitalization. However, the overall lack of attention to parents' mental well‐being underscored how the hospital environment primarily revolved around the medical care for the infant, often at the expense of the psychological needs of parents.

## 4 Discussion

The quantitative results of the survey reflected high overall parental satisfaction [23]. In this qualitative study, we explored parents' qualitative comments on family care in the NICU. While general expressions of satisfaction and praise were noted, the qualitative analysis focused on responses that included more elaborated comments, which could help enhance family care in NICUs. The main findings are expressed in the themes ‘Challenges in communication and recognition in NICU care’ and ‘Balancing family needs and support systems in NICU’. We found that parental insecurity, driven by fragmented communication, was a central concern for the parents. Such inadequacies undermined the trust that parents expected to develop with NICU staff—a trust that is foundational to FCC [28]. We found that the involvement of less experienced staff or trainees—especially without explanation—often triggered anxiety and distrust among parents. Another study describes that parents accept trainees in pediatric care when clear communication and supervision are in place to maintain trust [29]. Our findings similarly highlight the importance of transparency and professional conduct in hospital settings, where parental reassurance is closely tied to perceived competence in care [29]. Communication, therefore, needs to be not only consistent and clear but also individualized. Parents should be explicitly informed upon admission and regularly reminded that the hospital is a university teaching hospital where trainees participate in patient care.

Parents perceived elements of a partnership and mutual respect to be inconsistently enacted, often constrained by structural barriers such as delays in medication administration associated with time pressure. These challenges may be partly explained by the high clinical complexity and workload inherent to a level IV NICU with approximately 1200 admissions annually. Time constraints and competing clinical demands are a prevalent known challenge hindering the effective implementation of FCC in NICU settings, particularly in high‐acuity environments where technical and time‐sensitive care is prioritized [16, 30]. Furthermore, studies demonstrate a gap between professionals' perceived importance of FCC and actual delivery in practice, highlighting a misalignment between professional intent and enacted care [31]. This disconnect may contribute to parents' experiences of marginalization and exclusion during their infant's hospitalization. We found that the sense of exclusion was especially evident among parents of less critically ill infants, who often felt overlooked and underprioritized. Their accounts highlighted a perceived hierarchy of attention, where clinical urgency appeared to dictate the degree of emotional support and partnership extended. This experience challenges the principle of a partnership. It suggests that critiques of FCC often highlight its conditional application—where support depends on infant acuity rather than a consistent commitment to all families [32].

We found that parents reported a misalignment between their emotional needs and caring routines. Bonding opportunities like breastfeeding were frequently overlooked, reinforcing their perception of being positioned as observers rather than collaborators. While studies have found that individualized care planning has been shown to improve parental satisfaction and engagement [33], we identified that parents experienced standardized and task‐driven care. A study has found that in high‐acuity contexts, the prioritization of medical stabilization may constrain opportunities for parents' direct involvement in infant care, despite professionals acknowledging the importance of FCC [34], which highlights the importance of a continuous focus on holistic care that encompasses the whole trajectory, including post‐discharge. A further source of distress for parents was the expectation of active involvement without corresponding emotional or practical support. Accordingly, such dynamics risk conflating involvement with delegation, eroding the sense of collaboration that FCC is intended to foster [11].

The emotional and mental health needs of some parents were frequently perceived as neglected, with parents emphasizing the absence of private space as a key contributor to distress. The emotionally supportive environment of the NICU is crucial for both parental mental health and the parent–infant bond [35].

Overall, our findings underscore the need to embed humanization principles into NICU care. This means not only involving parents in care and treatment but also attending to their emotional realities, communicating transparently and offering consistent support, which requires appropriate training and education of healthcare professionals as well as adequate staffing levels. The findings should be interpreted in light of the study context of a level IV NICU with a long‐standing commitment to FCC and FICare, including unrestricted parental access, bedside accommodation, FCC and FICare training for newly hired nurses and NIDCAP certification. Despite these structures, parents reported unmet needs related to communication, clarity of roles and consistency in FCC practices. This suggests that structural support for FCC and FICare does not in itself ensure consistent parental experiences. When both interpersonal dynamics and structural barriers are addressed, FCC may evolve from a policy ideal to a holistic, lived experience—one where all parents, regardless of infant acuity, feel valued, heard and empowered as a family.

## 5 Strengths and Limitations

We found it a strength that we had many respondents and representation from both parents. Additionally, researcher triangulation was ensured through the diverse author group. During analysis, we chose to focus on responses that could gain insight into how family care could be improved in the NICU. A limitation was that only Danish‐speaking parents were able to respond to the questionnaire, as no other languages were offered in the questionnaire. Another limitation was the time of delivery of the questionnaires. The data were collected post‐discharge, possibly affecting recall due to emotional relief or memory gaps. However, we considered they offered insight into lasting impressions. Although the data is 6 years old, we still consider it applicable to contemporary NICU care as the field is still lacking consistency and standardization since no definitive standard for FCC has yet been established [13].

Parents particularly expressed a need for greater privacy, emotional support and predictable involvement in care and decision‐making. The transition to single‐family rooms in a new Childrens Hospital is expected to address privacy concerns and reduce exposure to other families' distressing experiences. In addition, structured parent peer‐support groups and professionally facilitated mother and father groups have been implemented. The unit has also begun introducing structured parent–professional conversations focused on individual parental needs. Ongoing initiatives include Family Infant Neurodevelopmental Education (FINE) certification for staff, the presence of International Board Certified Lactation Consultant (IBCLC)‐certified nurses, and weekly nutrition conferences to support feeding and breastfeeding. Future studies could evaluate the value or effectiveness of these interventions in terms of parents views on family care [25].

## 6 Implication for Clinical Practice

This article can inspire NICUs around the world to learn from evaluating parent satisfaction and experiences and these outcomes and findings. Parents have never been asked about their views and experiences concerning FCC in our NICU. This study highlights the areas for improvement in clinical practice to align with FCC principles and better meet parents' needs. We believed we were practicing FCC, but this study showed that recipients believed there were areas for improvement. The findings are an important reminder that practice does not necessarily reflect the principles that are assumed to be guiding care and that we continuously have to work on the FCC principles to adapt them in clinical practice.

## 7 Conclusions

Some parents' experiences of family care in the NICU do not fully align with the intended principles of FCC. Organizational priorities frequently conflict with FCC principles, especially in terms of communication and parental involvement. The findings underscore the need for improved communication strategies, better parental involvement in care decisions and more attention to emotional and relational support. Addressing these areas might enhance the overall NICU experience, meeting the needs of infants and families.

## Author Contributions

**Linea Grothe Larsen:** conceptualization; writing – original draft (lead); data curation (lead); formal analysis; methodology; writing – review and editing (equal). **Suzanne Forsyth Herling:** conceptualization; formal analysis; methodology; writing – original draft (supporting); writing – review and editing (equal). **Sanne Allermann Beck:** data curation; writing – review and editing (equal). **Anne Brødsgaard:** formal analysis (supporting); writing – review and editing (equal). **Jos M. Latour:** writing – review and editing (equal). **Rikke Louise Stenkjaer:** conceptualization; formal analysis; methodology; writing – original draft (supporting); writing – review and editing (equal).

## Funding

The authors have nothing to report.

## Conflicts of Interest

The authors declare no conflicts of interest.

## Data Availability

The data that support the findings of this study are available from the corresponding author upon reasonable request.
